# Cancer incidence trends in Baden-Württemberg (Southwest Germany) during and after the COVID-19 pandemic (2020–2023)

**DOI:** 10.1007/s00432-025-06349-w

**Published:** 2025-10-21

**Authors:** Lina Jansen, Silke Hermann, Susanne Bergbold, Volker Arndt

**Affiliations:** German Cancer Research Center (DKFZ), Epidemiological Cancer Registry Baden-Württemberg, Im Neuenheimer Feld 280, 69120 Heidelberg, Germany

**Keywords:** Cancer, COVID-19, Incidence, Germany, Trend, Cancer registry

## Abstract

**Purpose:**

While several countries reported an impact of the coronavirus disease (COVID-19) pandemic on cancer incidence in 2020, little is known about trends in the following years. This study examined changes in cancer incidence in Baden-Württemberg between 2015 and 2023.

**Methods:**

Data from the Baden-Württemberg Cancer Registry were used to calculate age-standardized and age-specific incidence rates for all cancers combined and for colorectal, lung, prostate, and breast cancer. Incidence rates for 2020 to 2023 were compared with those from a pre-pandemic reference period (2017–2019) and with expected rates based on modeled trends between 2015 and 2019 using standardized incidence ratios (SIRs).

**Results:**

Among men, the age-standardized overall cancer incidence declined significantly from 734.0 per 100,000 in 2019 to 672.9–681.7 during 2020–2023. In women, incidence declined from 542.2 in 2019 to 504.3–524.4, with statistically significant reductions in 2022 and 2023. Compared to 2017–2019 levels, 14,214 fewer cases (-5.5%) were diagnosed in 2020–2023; relative to model-based expectations, 19,525 fewer cases (-7.6%) were reported. Site-specific analyses showed significantly lower colorectal cancer incidence in both sexes from 2020 onwards (SIRs: 0.81–0.90). For men, part of this decline may reflect a pre-existing downward trend. No significant deviations were found for lung and prostate cancer. Female breast cancer incidence was significantly lower only in 2020 (SIR: 0.93).

**Conclusion:**

Cancer incidence in Baden-Württemberg remained consistently below pre-pandemic and expected levels from 2020 through 2023. Further research is warranted to disentangle potential contributing factors, including post-pandemic effects, competing mortality risks, and migration-related population changes.

**Supplementary Information:**

The online version contains supplementary material available at 10.1007/s00432-025-06349-w.

## Introduction

Restrictions and changes in healthcare access during the coronavirus disease (COVID-19) pandemic have globally influenced cancer incidence patterns. In many countries, cancer screening programs were suspended for several weeks starting in March or April 2020 (Mayo et al. 2021). Additionally, diagnosis of cancer might have been declined as a result of a decreased utilization of the health care system due to general contact avoidance and lockdown measures implemented during the pandemic. Conversely, press releases from stakeholders, for example in Germany from the German Cancer Research Center (DKFZ), the German Cancer Aid, and the German Cancer Society in April 2020, urged the public to promptly attend medical appointments to investigate suspicious symptoms, aiming to prevent delayed diagnoses and subsequent an increase in late-stage cancers at diagnosis (Deutsche Krebsgesellschaft (DKG) 2020).

Numerous studies from Germany (De Santis et al. 2023; Erdmann et al. 2024; Jansen et al. 2024; Kraywinkel et al. 2024; Voigtländer et al. 2023) as well as from other countries (Angelini et al. 2023) have reported declines in cancer incidence during 2020. In Germany, approximately 6.5% fewer cancer cases than expected were diagnosed (Kraywinkel et al. 2024). Following a sharp and abrupt decline in incidence rates in March and April 2020, rates increased again in the subsequent months. Nonetheless, in some countries and for certain cancer sites, incidence rates remained below expected levels in 2021 (Barclay et al. 2024; Burus et al. 2024; Garrido-Cantero et al. 2023; Howlader et al. 2025; Johansson et al. 2025). For instance during the second stringent lockdown in January 2021, in Bavaria, Germany, 25% fewer new cancer cases were reported than expected (Voigtländer et al. 2023). In Baden-Württemberg, while overall breast cancer incidence rates in 2021 were comparable to pre-pandemic levels, there was a notable 8% reduction in incidence among women aged 80 years and older compared to expected rates.

Despite these ongoing declines, there is a lack of publications on cancer incidence from 2022 onwards in comparison to pre-pandemic levels. The aim of this study was to investigate changes in age-standardized and age-specific incidence of total cancer as well as colorectal, lung, prostate, and breast cancer in the federal state of Baden-Württemberg (Germany) during the years 2020 to 2023 relative to the pre-pandemic period.

## Materials and methods

### Study population

Patients diagnosed with cancer (International Classification of Diseases (ICD)-10 codes C00 to C96, excluding C44 and C77-C79) between 2015 and 2023, residing in Baden-Württemberg at the time of diagnosis, were identified from the Baden-Württemberg Cancer Registry dataset (version: January 2025, epidemiological dataset). Patients notified by death certificate only (DCO) were included, with the year of death recorded as the year of diagnosis (4.1%). The district of Göppingen was excluded from the analysis due to known underreporting of cancer cases in recent years. For monthly analyses, patients with estimated months of diagnosis (1.1%) as well as DCO cases were excluded.

### General population

Data on the mid-year population of Baden-Württemberg by calendar year, sex, and age were obtained from the statistical offices of the German states (Statistisches Bundesamt). For analyses conducted by month, the population was assumed to be constant throughout the year due to the unavailability of monthly population data.

### Classification of variables

Site-specific analyses were performed for colorectal cancer (ICD-10: C18-C20), lung cancer (ICD-10: C33-C34), prostate cancer (ICD-10: C61), and female breast cancer (ICD-10: C50). These cancer sites were selected because they are the most frequently diagnosed both nationwide and in Baden-Württemberg, together accounting for 51% of all cases in 2023. Moreover, they provide sufficiently large sample sizes to enable robust annual trend analyses. In line with common practice in German cancer registry reporting, C33 and C34 were combined under the category of “lung cancer”, as tracheal cancers are very rare (0.05% of the C33-C34 group in 2023) and are routinely reported together with lung cancers. Age at diagnosis was categorized into the following groups: 0–49 years (with a further subdivision for total cancer into 0–39 and 40–49 years), 50–59, 60–69, 70–79, and 80 years and older. The calendar years 2017–2019 were designated as the pre-pandemic reference period, while 2020–2023 were considered pandemic years.

### Statistical methods

Age-standardized and age-specific incidence rates were calculated for each year and reported as cases per 100,000 person-years. Additionally, monthly age-standardized incidence rates were computed for the years 2019 to 2023. The population of Baden-Württemberg in 2020, stratified by the aforementioned age groups, was used as the standard population. For site-specific analyses, age-specific incidence rates were aggregated into two groups: 0–69 years and 70 years and older, with age-standardization performed within each group.

Two approaches were employed to estimate the expected incidence rates and case numbers for the years 2020 to 2023. In the first approach, age-specific incidence rates from 2017 to 2019 served as the reference period. Observed age-specific and age-standardized incidence rates from 2020 to 2023 were compared to this reference using standardized incidence ratios (SIRs). Additionally, the age-specific incidence rates from the reference period were applied to the corresponding population figures in subsequent years to calculate the expected number of cases. The second approach assumed that incidence trends observed from 2015 to 2019 would continue into the following years. For each cancer site and age group, a Poisson regression model was fitted with case counts as the outcome, calendar year as the explanatory variable, and the logarithm of person-years as an offset. The SIRs and 95% confidence intervals for the year variable are provided in Supplementary Table 1. Using the model coefficients, expected age-specific incidence rates (with standard errors) and expected case numbers for 2020 to 2023 were predicted. Age-standardized expected incidence rates were then derived as weighted sums of the age-specific estimates. Comparisons between observed and expected incidences were conducted using SIRs.

### Comparative incidence data from other federal states and countries

To compare trends in age-standardized cancer incidence in Baden-Württemberg with those in other German federal states and countries, age-standardized incidence data (standardized to the European Standard Population, 1976) for total cancer were obtained from publicly available sources. Details regarding data sources, methods, and results are provided in the supplement.

### Statistical analyses: General aspects

A p-value < 0.05 was considered statistically significant. No adjustments for multiple comparisons were performed. Statistical analyses were performed using SAS Enterprise Guide V9.4 (SAS Institute Inc., Cary, NC, USA), R (version 4.4.1), and RStudio (version 2024.09.0 + 375), using the tidyverse, patchwork, and ggpubr packages.

## Results

### Total cancer

**Fig. 1:**
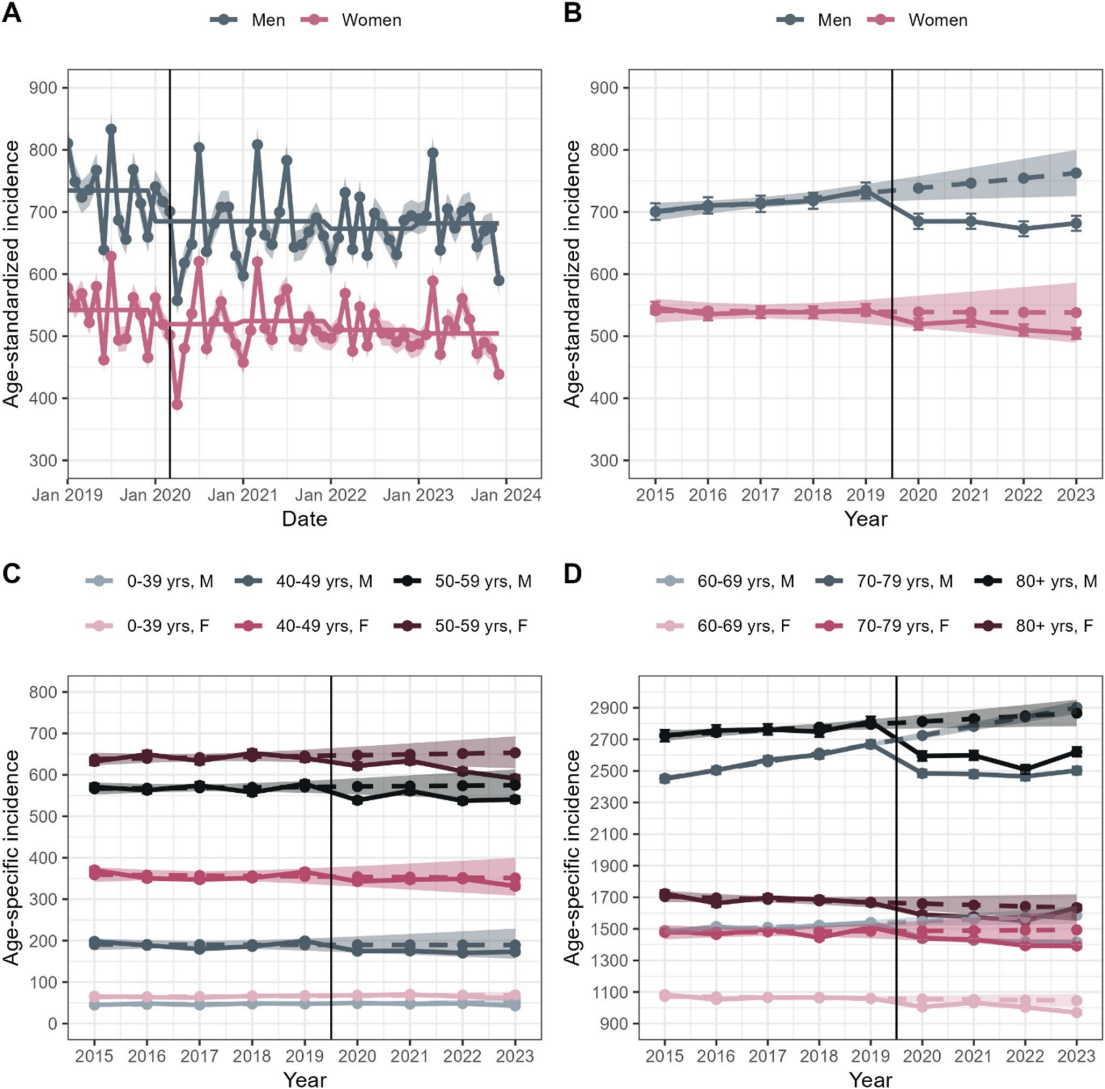
Age-standardized total cancer incidence by month (**A**), and sex-stratified total cancer incidence with modelled/predicted incidence based on age-specific trends between 2015 and 2019, shown as age-standardized (**B**) and age-specific incidence: 0–59 years (**C**) and 60 years and older (**D**). Observed estimates are shown as solid lines with standard errors represented by error bars. Modelled/predicted estimates are shown as dashed lines with 95% confidence intervals indicated by shaded bands

Age-standardized total cancer incidence in men rose from 700.6 to 734.5 per 100,000 between 2015 and 2019 (Fig. 1). During the pandemic (2020–2023), incidence declined significantly to 672.9–685.0 per 100,000, with SIRs of 0.95 (2021), 0.93 (2022), and 0.94 (2023) when compared to the years 2017–2019 as reference (Table 1). Compared to projections based on pre-pandemic trends, SIRs were even lower (0.92, 0.89, and 0.89) (Supplementary Table 2). Age-stratified analyses revealed that a decline was observed in men aged 40 and older. In contrast, incidence in men under 40 was higher in 2020 (SIR 1.06) but dropped below expected levels by 2023 (SIR 0.91). Overall, the observed patterns closely aligned with those predicted by the projections.

**Table 1:** Comparison of observed age-standardized and age-specific cancer incidence during the pandemic years with expected incidence based on rates from the 2017–2019 reference period

Site	Sex	Age	Standardized incidence ratio (95% confidence interval) between the specific year and the reference			
			2020	2021	2022	2023
Total	Male	0–39	1.06* (1.00–1.13)	1.00 (0.94–1.07)	1.03 (0.96–1.10)	0.91** (0.85–0.98)
		40–49	0.93* (0.87–0.99)	0.93* (0.87–1.00)	0.90** (0.85–0.97)	0.92* (0.86–0.98)
		50–59	0.94*** (0.91–0.98)	0.98 (0.95–1.02)	0.94*** (0.91–0.97)	0.95** (0.92–0.98)
		60–69	0.95*** (0.93–0.97)	0.94*** (0.92–0.96)	0.93*** (0.91–0.96)	0.93*** (0.91–0.95)
		70–79	0.95*** (0.93–0.97)	0.95*** (0.93–0.97)	0.94*** (0.92–0.96)	0.96*** (0.94–0.98)
		80 +	0.93*** (0.91–0.96)	0.94*** (0.91–0.96)	0.90*** (0.88–0.93)	0.94*** (0.92–0.97)
		All	0.95* (0.91–0.99)	0.95* (0.91–0.99)	0.93*** (0.90–0.97)	0.94** (0.91–0.98)
	Female	0–39	1.04 (0.98–1.10)	1.07* (1.01–1.14)	0.99 (0.94–1.05)	0.93* (0.87–0.98)
		40–49	0.97 (0.92–1.01)	0.98 (0.93–1.03)	0.98 (0.94–1.03)	0.93** (0.89–0.98)
		50–59	0.97* (0.94–1.00)	0.99 (0.96–1.02)	0.95*** (0.92–0.98)	0.92*** (0.89–0.95)
		60–69	0.95*** (0.92–0.97)	0.97* (0.94–1.00)	0.94*** (0.92–0.97)	0.91*** (0.89–0.94)
		70–79	0.97* (0.94–1.00)	0.97** (0.94–0.99)	0.94*** (0.91–0.96)	0.94*** (0.91–0.96)
		80 +	0.94*** (0.92–0.97)	0.94*** (0.91–0.96)	0.92*** (0.90–0.95)	0.97* (0.95–1.00)
		All	0.96 (0.92–1.00)	0.97 (0.93–1.01)	0.94** (0.91–0.98)	0.93** (0.90–0.97)
Colorectal	Male	0–69	0.92 (0.81–1.05)	0.92 (0.81–1.04)	0.89 (0.78–1.01)	0.89 (0.78–1.01)
		70 +	0.88*** (0.82–0.95)	0.84***(0.78–0.90)	0.80*** (0.74–0.86)	0.80***(0.74–0.86)
		All	0.90 (0.79–1.01)	0.87* (0.77–0.98)	0.83**(0.74–0.94)	0.84**(0.74–0.95)
	Female	0–69	0.95 (0.82–1.11)	0.87 (0.74–1.01)	0.87 (0.75–1.02)	0.83* (0.71–0.97)
		70 +	0.82 ***(0.76–0.89)	0.85***(0.79–0.91)	0.83*** (0.77–0.90)	0.80*** (0.75–0.87)
		All	0.87 (0.77–1.00)	0.86* (0.75–0.98)	0.85* (0.74–0.97)	0.81** (0.71–0.93)
Lung	Male	0–69	0.96 (0.84–1.10)	0.91 (0.79–1.05)	0.87* (0.76–1.00)	0.87 (0.76–1.00)
		70 +	0.98 (0.91–1.05)	0.96 (0.89–1.04)	0.92* (0.85–0.99)	0.94 (0.88–1.02)
		All	0.97 (0.86–1.10)	0.94 (0.83–1.07)	0.90 (0.79–1.02)	0.91 (0.80–1.03)
	Female	0–69	1.04 (0.89–1.21)	0.99 (0.85–1.16)	0.98 (0.84–1.15)	0.94 (0.80–1.10)
		70 +	1.13*(1.03–1.24)	1.18*** (1.08–1.29)	1.19*** (1.09–1.31)	1.27*** (1.16–1.38)
		All	1.08 (0.93–1.24)	1.08 (0.93–1.24)	1.08 (0.93–1.24)	1.08 (0.94–1.25)
Prostate	Male	0–69	0.95 (0.86–1.04)	1.00 (0.91–1.10)	1.04 (0.95–1.13)	1.07 (0.98–1.17)
		70 +	0.94* (0.90–0.99)	1.01 (0.97–1.06)	1.03 (0.98–1.07)	1.08*** (1.04–1.13)
		All	0.94 (0.87–1.02)	1.01 (0.93–1.09)	1.03(0.95–1.11)	1.08 (1.00–1.16)
Breast	Female	0–69	0.92* (0.86–0.98)	1.00 (0.94–1.07)	0.95 (0.89–1.02)	0.94 (0.88–1.01)
		70 +	0.94* (0.90–1.00)	0.94* (0.89–0.99)	0.93** (0.88–0.98)	0.98 (0.93–1.03)
		All	0.93 *(0.87–0.99)	0.98 (0.92–1.05)	0.94 (0.88–1.01)	0.96 (0.90–1.02)

Significant values (*p* < 0.05) are shown in bold. Levels of significance are additionally indicated by asterisks: *** *p* < 0.001, ** *p* < 0.01, * *p* < 0.05

In women (Fig. 1), age-standardized total cancer incidence was stable between 2015 and 2019 (535.6–543.2 per 100,000) but declined afterwards (504.3–524.4). Because of this pre-pandemic stability, only comparisons with the reference period are reported (Table 1, Supplementary Table 2). The decline was significant in 2022 (SIR 0.94) and 2023 (SIR 0.93). Age-specific trends resembled those in men for women aged 50 and older. In women aged 40–49, decreases were smaller and not significant in 2020–2022. For those under 40, incidence tended to be higher in 2020 (1.04) and 2021 (1.07) but was significantly lower in 2023 (0.93).

Monthly data showed a drop in April 2020 but no clear pattern afterwards, despite overall lower rates (Fig. 1). From 2020 to 2023, 258,157 cases were recorded (Supplementary Table 3), with 14,214 and 19,525 cases less than expected by reference and projected incidences.

### Colorectal cancer

**Fig. 2:**
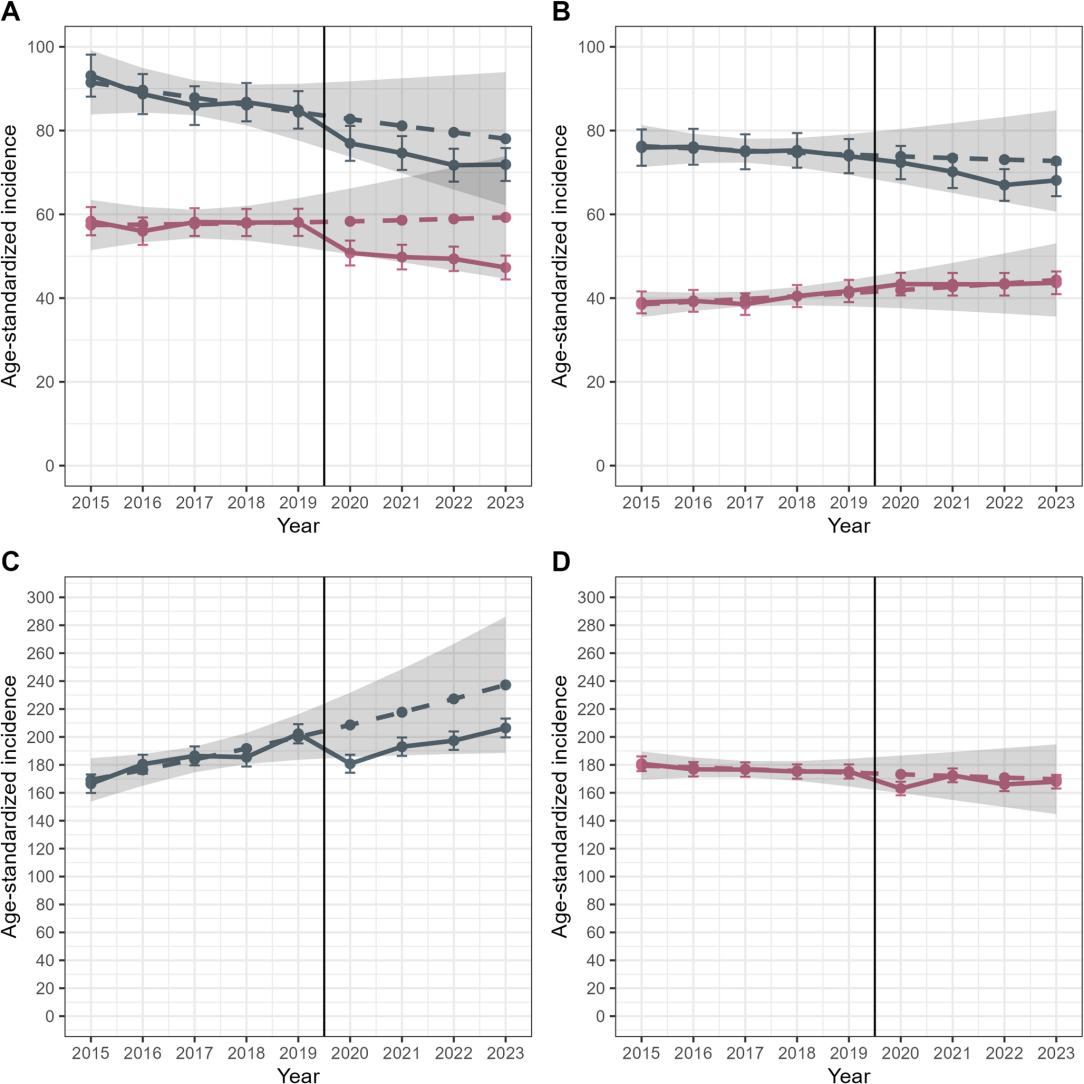
Age-standardized cancer incidence per 100,000 person-years by sex (women in pink, men in gray) for colorectal (**A**), lung (**B**), prostate (**C**), and breast cancer (**D**). Observed estimates are shown as dashed lines with standard errors represented by error bars, while modelled/predicted estimates are shown as solid lines with 95% confidence intervals indicated by shaded bands

**Fig. 3:**
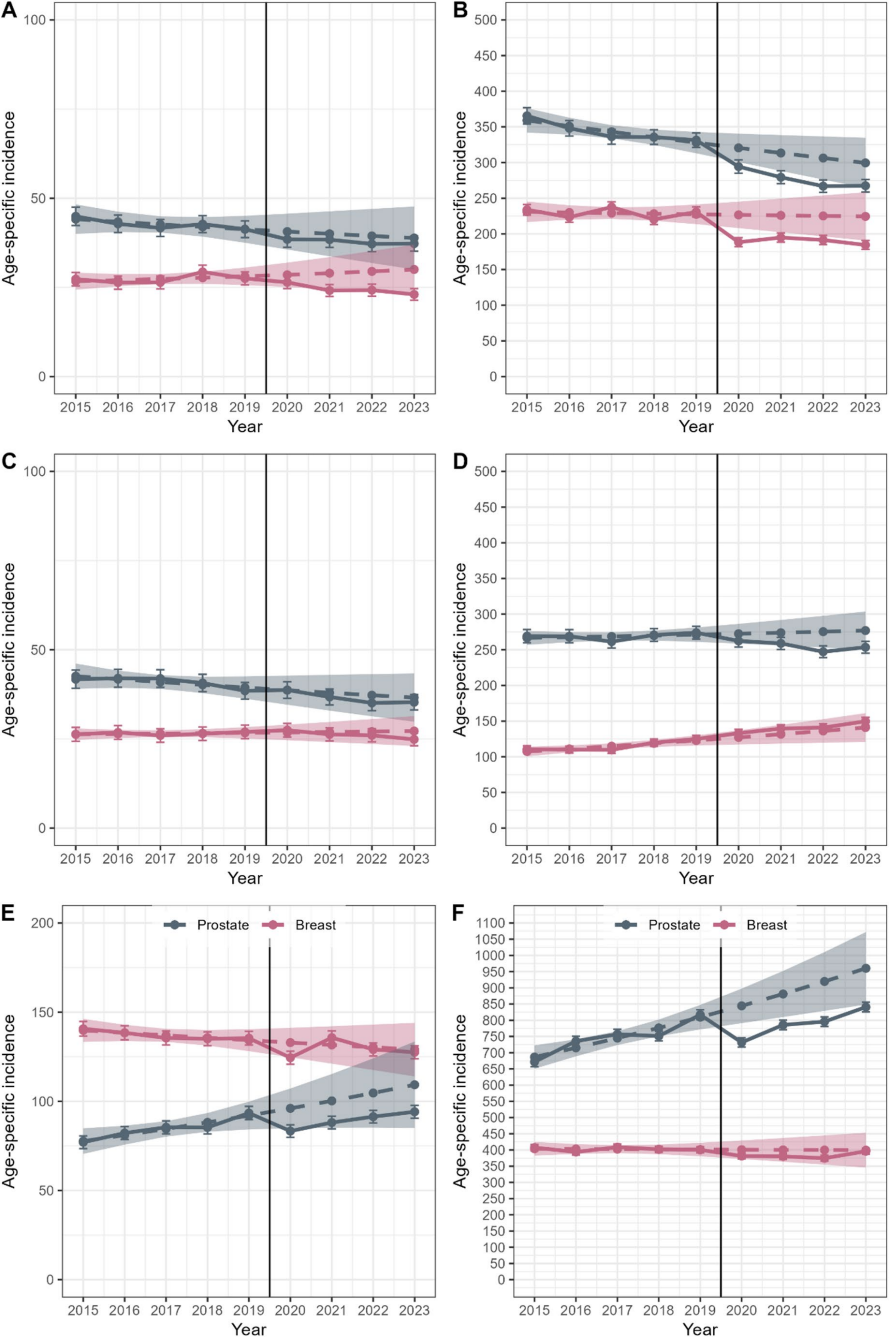
Age-specific cancer incidence by sex (women in pink, men in gray), based on trends from 2015 to 2019, for colorectal cancer (**A**: 0-69 years; **B**: 70 +), lung cancer (**C**: 0-69; **D**: 70 +), and prostate and breast cancer (**E**: 0–69; **F**: 70 +). Observed estimates are shown as dashed lines with standard errors represented by error bars, while modelled/predicted estimates are shown as solid lines with 95% confidence intervals indicated by shaded bands

Age-standardized colorectal cancer incidence in men declined from 93.1 per 100,000 in 2015 to 84.9 in 2019 (Fig. 2), with a further decrease to 77.0 in 2020 and 71.9 in 2023. Compared to the reference, the decrease was significant from 2021 onwards (SIRs: 0.83–0.87, Table 1). When compared to projections, SIRs ranged from 0.90 to 0.93 and were no longer statistically significant (Supplementary Table 2). In women, incidence remained stable between 2015 and 2019 (56.0–58.4) but decreased to 47.3–50.8 thereafter, with significant reductions from 2021 to 2023 (SIRs 0.81–0.86). These reductions lost significance when compared to projections but remained of similar magnitude. Age-specific analyses showed that declines from 2020 onwards were more pronounced in those aged 70 and older for both sexes (Fig. 3, Table 1, Supplementary Table 2).

Monthly analyses revealed no clear patterns in colorectal cancer incidence (Supplementary Fig. 1). Between 2020 and 2023, 26,701 colorectal cancer cases were diagnosed (Supplementary Table 3), representing 4,533 (reference) and 3,496 (projection) fewer cases than expected.

### Lung cancer

In men, age-standardized lung cancer incidence declined from 75.9 per 100,000 in 2015 to 73.9 in 2019 and further to 68.1 by 2023 (Fig. 2). However, differences during the pandemic years (2020–2023) were not statistically significant compared to the reference (SIRs: 0.90–0.97) or the projection (0.92–0.98). Age-specific analyses showed significantly lower incidence in 2022 for both age groups (0–69 years: SIR 0.87; 70 + years: 0.92). The reduction remaining significant among older men when compared to projections.

In women, incidence rose from 39.0 in 2015 to 41.7 in 2019 and remained stable at 43.3–43.7 between 2020 and 2023. No significant differences between pandemic and pre-pandemic years were found when compared to the reference period (all SIRs 1.08) or projections (0.98–1.03). Among women aged 70 + , incidence was elevated in all pandemic years compared to the reference (SIRs: 1.13–1.27), but this difference diminished and lost significance when accounting for pre-pandemic trends (SIRs: 1.03–1.06).

Monthly incidence analyses showed no clear patterns (Supplementary Fig. 1). In total, 24,294 lung cancer cases were diagnosed from 2020 to 2023—329 and 709 fewer than expected based on the reference and the projections, respectively (Supplementary Table 3).

### Prostate cancer

Age-standardized prostate cancer incidence increased from 166.5 to 202.3 per 100,000 men between 2015 and 2019 (Fig. 2). In 2020, it declined to 180.8 but rose again to 206.4 by 2023. Compared to the reference period, no significant differences were observed with SIRs between 0.94 in 2020 and 1.08 in 2023 (Table 1). Assuming a continued pre-pandemic increase, incidence was lower in all pandemic years (SIRs 0.87–0.89), with a significant reduction only in 2020 (Supplementary Table 2).

Age-specific analyses showed significant reductions in incidence compared to the reference in 2020 (0.94) and 2023 (1.08) among men aged 70 + . When considering projected trends, SIRs declined in both age groups, with lower incidence among younger men in 2020 (SIR 0.87) and among older men in all pandemic years (SIRs 0.87–0.89).

Monthly data revealed a marked drop in incidence between April and June 2020 (Supplementary Fig. 1). From 2020 to 2023, 39,264 prostate cancer cases were diagnosed—647 more than expected based on the reference, but 5,788 fewer than projected (Table 3).

### Breast cancer

Age-standardized breast cancer incidence declined from 180.8 per 100,000 in 2015 to 175.3 in 2019. In 2020, it dropped to 163.1 and fluctuated thereafter—rising to 172.5 in 2021, then falling to 166.0 in 2022 and 167.9 in 2023 (Fig. 2). Compared to the reference, only 2020 showed a significant decrease (SIR 0.93, Table 1). Differences were slightly smaller and not significant when compared to projections (Supplementary Table 2).

Among women aged 70 + , incidence was significantly lower in 2020–2022 compared to the reference (SIRs 0.93–0.94), and in all four years when compared to projections (SIRs 0.87–0.89). For younger women, significant differences were observed only in 2020 (reference: 0.92; projection: 0.87).

Monthly analyses showed a marked drop in April 2020 (Supplementary Fig. 1). Between 2020 and 2023, 38,484 breast cancer cases were diagnosed—1,934 and 1,014 fewer than expected based on the reference and projections, respectively (Supplementary Table 3).

## Discussion

Cancer incidence declined in both men and women with the onset of the COVID-19 pandemic in Baden-Württemberg in 2020. Contrary to expectations of a rebound, incidence remained lower through 2023, resulting in 14,214 (reference) to 19,525 (projection) fewer cases in 2020 to 2023. The decline was not limited to older adults. Site-specific analyses revealed the largest reductions in colorectal cancer among the elderly, partly reflecting a pre-existing decline in men. No consistent pattern was observed for lung cancer. Prostate cancer incidence dropped in 2020 and remained lower in older men under continued pre-pandemic trend assumptions. Breast cancer incidence fell sharply in April 2020 but recovered thereafter.

Reductions in cancer incidence during 2020 have been reported in many countries, including the Nordic countries (Johansson et al. 2025), the Netherlands (Eijkelboom et al. 2023), Poland (Choręza et al. 2024), the United Kingdom (Barclay et al. 2024), Northern Ireland (Bennett et al. 2024), Spain (Garrido-Cantero et al. 2023), and the United States (Burus et al. 2024). The magnitude and post-2020 trends varied substantially. For example, among the Northern Countries, incidence was between 2.3% (Norway) and 7.6% (Sweden) lower in 2020 (Johansson et al. 2025). While Sweden and Finland saw continued reductions in 2021 (-3.6% and -2.8%), Denmark and Norway returned to pre-pandemic levels. The size of our reduction in 2020 (-5% in men and -4% in women) aligns with this range and is slightly smaller than the -6.5% reported for Germany  (Kraywinkel et al. 2024). Unlike the Nordic countries, our data show persistent reductions in 2021 (-5% in men, -3% in women). For later years, publications are scarce: Based on clinical registry data, prostate cancer incidence returned to pre-pandemic levels in Sweden in 2022 (Zaurito et al. 2025), and stable lung cancer rates were observed in parts of Northern Poland (Romaszko-Wojtowicz et al. 2024). In contrast, we observed significantly lower than expected incidence based on the reference period in 2023 for total cancer (− 6% in men and − 7% in women) and colorectal cancer (− 12% in men and − 19% in women). Adjusting for pre-pandemic trends resolved statistical significance for all subgroups except for total cancer in men, though reductions larger than 5% persisted across most cancer sites and sexes, except female lung and breast cancer.

Owing to limited publications for recent years, we examined publicly available cancer registry data on total cancer incidence from 2015 to the latest year available (see Supplementary Methods, Supplementary Table 5, Supplementary Figs. 2 and 3). To aid interpretation of differences across German federal states, a map of the states is provided in Supplementary Fig. 4. Baden-Württemberg, the third largest federal state (2023: 11.2 million inhabitants), is located in the south-west of Germany. The state has an average primary care physician density, with 1,272 inhabitants per physician (Germany: 1,264) in 2024 (Statistisches Bundesamt 2025), but the lowest hospital bed density (4.7 per 1,000 inhabitants) of all states in 2023 (Statistics Office 2023). Baden-Württemberg has good coverage of mammography screening, with 10 certified units across 39 locations (Referenzzentrum Mammographie SüdWest 2025). With three comprehensive cancer centers, it also has an excellent cancer care infrastructure (Netzwerk Onkologische Spitzenzentren 2025). Smoking prevalence (Deutsches Krebsforschungszentrum and Deutsche Krebshilfe 2025) is lower than the national average: 21.8% in men (range across states: 20.9–29.0%) and 13.9% in women (13.9–19.5%). Socioeconomic deprivation is the lowest in Germany, comparable to the neighboring state Bavaria (Michalski et al. 2022). Total cancer incidence trends varied across federal states: some, such as Hamburg and Schleswig–Holstein, showed little change from pre-pandemic levels during 2020–2022, while others, for example North Rhine-Westphalia and Lower Saxony, mirrored the declines observed in Baden-Württemberg. In 2022, incidence was lower than pre-pandemic across all federal states, though reductions were modest. International patterns were similarly heterogeneous. Following the decline in 2020, Belgium showed slightly elevated incidence in 2021 and 2022, while most countries showed comparable to slightly lower than pre-pandemic levels in 2022. Only the Netherlands and Norway provided 2023 data. In both countries, estimates for 2023 were lower than 2022, though data may be incomplete due to reporting delays. In summary, the findings highlight substantial variation in pandemic-related effects both between and within countries, underscoring the need for regional surveillance of potential long-term impacts on cancer incidence.

Reductions in cancer incidence might be related to participation in screening programs during the pandemic. The organized mammography screening program was paused for four to six weeks starting in April 2020 (Kooperationsgemeinschaft Mammographie 2022). After the pause of the program, screening units extended their opening hours and made up for more than half of the missed screenings in 2020. In 2021, the screening invitation (97%) and participation rate (51%) were slightly above average (Kooperationsgemeinschaft Mammographie 2023) and were on pre-pandemic levels in 2022 (Kooperationsgemeinschaft Mammographie 2024). Our observed incidences fit to these developments, showing a strong decline during the pause of the screening program followed by a stabilization around pre-pandemic levels. Colorectal cancer screening by blood test and colonoscopy contributed to a decrease in cancer incidence over the last years (Brenner et al. 2016). In 2019, an invitation process was started, which increased participation rates. Official participation rates are only available for 2021 and 2022 showing a constant to slightly increasing participation rate (Gesundheitsforen Leipzig GmbH Evaluationsbericht Darmkrebs 2024). The overall number of screening colonoscopies increased continuously from 2019 to 2023 (Mangiapane et al. 2021a, b). Thus, ours results of reduced colorectal cancer incidence up to 2023 cannot be explained by reduction in screening participation. Prostate cancer screening is not organized in Germany and the costs for prostate specific antigen test (PSA test) are not covered by the statutory health insurance. Therefore, there is no data on changes of participation rates over time. We saw a strong upward trend prior to the pandemic. If it had been continued after 2019, our observed post-pandemic incidence would be lower than expected. Skin cancer screening were markedly reduced in 2020 (-20.3%) and 2021 (− 10.3%) and started to slightly develop towards pre-pandemic levels over the years 2022 and 2023. This reduction will contribute to the lower total cancer incidence levels after the pandemic. Overall, the changes in screening participation rates might probably contribute much to the reduction in 2020 but less to the still lower cancer incidence in 2022 and 2023.

Age-specific analyses showed that reductions in cancer incidence were not confined to the elderly, who face the highest risk of severe COVID-19, but also affected individuals from their 40 s. Relative reductions were similar across ages, while absolute decreases increased with age. In the Nordic countries, no reductions were seen among those aged 18–49, except in Sweden (− 4.0%), with the largest declines typically in the age group 50–69 (Johansson et al. 2025). Thus, reductions were not limited to screening ages but also reflect reduced healthcare utilization.

Additional factors potentially influencing cancer incidence during and after the COVID-19 pandemic include migration and competing mortality risks due to COVID-19-related deaths. Following the Russian invasion of Ukraine in 2022, the number of refugees in Baden-Württemberg rose significantly. Net migration from other countries to the region reached + 205,210 individuals in 2022 and + 102,025 in 2023, compared to + 55,147 in 2019 (Statistisches Landesamt Baden-Württemberg). Refugees from Ukraine tend to be younger, generally well-educated, and in sufficiently good health to undertake the journey to Germany (Décieux and Ette 2024). Although all refugees are entitled to medical care in Germany, language barriers and limited familiarity with the healthcare system may hinder timely cancer diagnoses. The population increase due to migration in 2022 and 2023 corresponds to 1.8% and 0.9% of the total population of Baden-Württemberg, respectively. This demographic shift may have contributed to a slight reduction in cancer incidence. Moreover, COVID-19-related deaths also altered population dynamics during the pandemic. Given that cancer and severe COVID-19 outcomes share common risk factors, such as smoking and obesity (Mahamat-Saleh et al. 2021), a competing risk scenario may have occurred: individuals at higher risk for both conditions might have died from COVID-19 before a cancer diagnosis could be made. This could have led to a transient decrease in cancer incidence in subsequent years. However, the overall number of COVID-19-related deaths in Baden-Württemberg remained relatively low, with 20,411 reported by the end of 2023 (Robert Koch Institut 2025). In summary, while migration and competing mortality risks may have contributed to the observed decline in cancer incidence, their overall impact is likely to be modest.

The strengths of this study include the use of high-quality cancer registry data covering a large geographic area. The analysis also encompasses all post-pandemic years through 2023 and takes pre-pandemic trends into account. Limitations include the lack of long-term pre-pandemic trend data and limited statistical power in the comparison between observed incidence rates and model-based estimates. An additional potential limitation is a slight underestimation of cancer incidence in the most recent years due to delays in physician reporting. However, internal data checks, such as analyses by region and trends in the number of reports by reporting sources, do not indicate any major issues. A comparison of age-standardized incidence rates of total cancer based on the original analysis dataset and an updated dataset from June 2025 revealed a 1.2% higher incidence in men and a 1.4% higher incidence in women in the updated dataset. Another potential source of slight underestimation of cancer incidence, particularly in the most recent years, is the use of population estimates based on the 2011 Census. The 2022 Census revealed that the actual population is smaller than previously assumed (Statistisches Bundesamt 2024). However, updated population data are not yet available.

In summary, our study demonstrates a continued and unexpectedly lower cancer incidence compared to pre-pandemic levels, both for overall cancer and for specific cancer sites. Between 2020 and 2023, an estimated 14,214 (relative to pre-pandemic levels) to 19,525 (relative to projections) fewer cases were diagnosed than expected. The reduction in incidence was not limited to older individuals or to those within screening age groups. Although true declines in cancer incidence are desirable, it is essential to rule out the possibility that cases have been missed and may be diagnosed later at more advanced stages. Continued monitoring of cancer incidence, including stage at diagnosis, is therefore essential. In addition, more detailed site-specific analyses are needed to improve understanding of the observed decreases.

## Supplementary Information

Below is the link to the electronic supplementary material.

Supplementary Material 1

## Data Availability

The data that support the findings of this study are available from the Baden-Württemberg Cancer Registry but restrictions apply to the availability of these data, which were used under license for the current study, and so are not publicly available. Data are however available from the authors upon reasonable request and with permission of the Baden-Württemberg Cancer Registry.
