# Supplementary material for: Cancer incidence trends in Baden-Württemberg (Southwest Germany) during and after the COVID-19 pandemic (2020–2023)
Source: J Cancer Res Clin Oncol. 2025 Oct 21;151(12):300. doi: 10.1007/s00432-025-06349-w (PMC12540955; doi:10.1007/s00432-025-06349-w)

Cancer Incidence Trends in Baden-Württemberg (Southwest Germany) During and After the COVID-19 Pandemic (2020-2023)

Supplementary Files

Lina Jansen[1], Silke Hermann[1], Susanne Bergbold[1], Volker Arndt[1]

[1] Epidemiological Cancer Registry Baden-Württemberg, German Cancer Research Center (DKFZ), Heidelberg, Germany

Corresponding author:

Lina Jansen, PhD
Epidemiological Cancer Registry Baden-Württemberg
German Cancer Research Center (DKFZ)
Im Neuenheimer Feld 280
69120 Heidelberg
Germany
l.jansen@dkfz-heidelberg.de

Supplementary methods

We searched the internet for publicly available cancer registry websites from other German federal states and European countries that provide age-standardized (European Standard Population 1976) incidence estimates for total cancer (ICD-10 C00-C97, excluding C44), stratified by sex, for the years 2015 through at least 2021. Data were available for all western German federal states except Rhineland-Palatinate and Hesse. Eastern German states were excluded due to structural changes in cancer registry operations after 2015.

At the European level, relevant incidence data were retrieved from the Netherlands, Belgium, and the NordCan project, restricted to the larger countries (Denmark, Finland, Iceland, Norway, and Sweden).

Age-standardized total cancer incidence estimates were extracted and presented graphically. For Baden-Württemberg, estimates were calculated from the study dataset. To compare pre-pandemic levels with later years, standardized incidence ratios (SIRs) were computed using the average incidence in 2017-2019 as the reference and comparing it to each year from 2020

onwards. As the external databases do not report standard errors or confidence intervals, formal statistical testing was not possible.

Due to varying data update schedules across registries, reporting delays – especially in recent years – may result in underestimation of cancer incidence. For example, the 2023 estimate from the Netherlands is marked as a provisional figure. Data sources for all extracted estimates are listed in Supplementary Table 4. Data were accessed on February 27, 2025.

Supplementary result – National and international comparisons

Supplementary Figure 2 presents trends in age-standardized total cancer incidence for men and women across German federal states. A decline in incidence in 2020 compared to the reference period (2017-2019) was observed in most but not all states, with standardized incidence ratios (SIRs) ranging from 0.94 in North Rhine-Westphalia to 0.98 in Schleswig-Holstein (see Supplementary Table 5). In several states, incidence remained below pre-pandemic levels in the following years. However, differences in pre-pandemic trends limit comparability across states.

Internationally, substantial variation in trends was also observed (Supplementary Figure 3). All examined countries reported lower cancer incidence among men in 2020 compared to 2017-2019, with SIRs ranging from 0.91 in Sweden to 0.99 in Iceland. In subsequent years, incidence largely returned to pre-pandemic levels or followed expected trends. Similar to Baden-Württemberg, the Netherlands and Norway – the only countries providing data for 2023 – showed a decrease in incidence, though this may reflect delayed reporting rather than a true decline.

Among women, cancer incidence in 2020 was also lower in most German states, with the exception of the northern states of Bremen, Hamburg, and Schleswig-Holstein. SIRs ranged from 0.91 in Saarland to 0.98 in Lower Saxony. In later years, some states, such as Bavaria and North Rhine-Westphalia, exhibited patterns similar to those observed in Baden-Württemberg, with sustained lower incidence. However, trends varied widely between states.

International data for women also showed declines in 2020 (SIRs: 0.93 in the Netherlands to 0.99 in Iceland), followed by a return to expected levels in most countries. Again, lower incidence in 2023 in the Netherlands and Norway may be due to incomplete case ascertainment.

Overall, while most regions reported a notable decline in cancer incidence in 2020, both the magnitude of the decline and post-pandemic trends varied substantially. Notably, no indication of a catch-up in missed cases from 2020 was observed in any region.

Supplementary Table 1 Annual change of cancer incidence (standardized incidence ratio and 95% confidence interval) in Baden-Württemberg in 2015-2019 estimated using Poisson regression

Site	Sex	Age group				
		0-39/0-49[a]<br>40-49	50-59	60-69	70-79	80+
Total	Male	1.01 (0.99 - 1.04)<br>1.00 (0.97 - 1.03)	1.00 (0.99 - 1.01)	1.01 (1.00 - 1.01)	**1.02 (1.02 - 1.02)***	1.01 (1.00 - 1.01)
Total	Female	1.01 (0.99 - 1.03)<br>1.00 (0.98 - 1.02)	1.00 (0.99 - 1.01)	1.00 (0.99 - 1.00)	1.00 (0.99 - 1.01)	0.99 (0.99 - 1.00)
Colorectal	Male	0.97 (0.91 - 1.03)	1.01 (0.99 - 1.03)	0.97 (0.95 - 0.99)	**0.97 (0.97 - 0.98)****	0.98 (0.96 - 1.00)
Colorectal	Female	0.97 (0.90 - 1.05)	**1.06 (1.02 - 1.09)***	1.00 (0.98 - 1.01)	1.01 (0.99 - 1.03)	0.99 (0.96 - 1.01)
Lung	Male	0.97 (0.90 - 1.05)	0.96 (0.92 - 1.01)	0.99 (0.98 - 1.00)	1.02 (1.00 - 1.04)	0.98 (0.97 - 1.00)
Lung	Female	**0.96 (0.94 - 0.97)****	1.00 (0.98 - 1.02)	1.01 (1.00 - 1.03)	**1.05 (1.03 - 1.06)***	1.01 (0.98 - 1.05)
Breast	Female	0.99 (0.97 - 1.00)	0.99 (0.98 - 1.00)	0.99 (0.98 - 1.01)	0.99 (0.97 - 1.01)	1.01 (0.98 - 1.04)
Prostate	Male	0.96 (0.89 - 1.03)	**1.03 (1.01 - 1.06)***	**1.05 (1.03 - 1.07)***	**1.06 (1.04 - 1.08)****	1.02 (0.99 - 1.04)

Significant values ($p < 0.05$) are shown in bold. Levels of significance are additionally indicated by asterisks: *** $p < 0.001$, ** $p < 0.01$, * $p < 0.05$.

[a] For total cancer, the age group 0-49 was split up to 0-39 and 40-49 years. Therefore, two estimates are shown. For the other cancer sites, the sample size was too small to model a trend for persons aged up to 39 years.

Supplementary Table 2 Comparison of the observed age-standardized and age-specific cancer incidence (per 100.000 person-years) in the pandemic years versus the estimated incidence in the pandemic years based on the short-term trend between 2017 and 2019 (Projection)

Site	Sex	Age	Standardized incidence ratio (95% confidence interval) between the specific year and the projected incidence in this year			
			2020	2021	2022	2023
Total	Male	0-39	1.03 (0.93 - 1.14)	0.96 (0.85 - 1.08)	0.97 (0.84 - 1.12)	0.86 (0.72 - 1.01)
		40-49	0.92 (0.82 - 1.04)	0.93 (0.80 - 1.07)	0.90 (0.76 - 1.07)	0.92 (0.75 - 1.12)
		50-59	**0.94** (0.90 - 0.99)**	0.98 (0.93 - 1.03)	**0.94* (0.88 - 1.00)**	0.94 (0.88 - 1.01)
		60-69	**0.93*** (0.91 - 0.96)**	**0.91*** (0.89 - 0.94)**	**0.90*** (0.87 - 0.93)**	**0.89*** 0.86 - 0.93)**
		70-79	**0.91*** (0.89 - 0.93)**	**0.89*** (0.87 - 0.91)**	**0.87*** (0.85 - 0.89)**	**0.86*** 0.84 - 0.88)**
		80+	**0.92*** (0.90 - 0.95)**	**0.92*** (0.89 - 0.95)**	**0.88*** (0.85 - 0.91)**	**0.91*** 0.88 - 0.95)**
		All	**0.93*** (0.89 - 0.97)**	**0.92*** (0.87 - 0.96)**	**0.89*** (0.84 - 0.94)**	**0.89*** (0.84 - 0.95)**
	Female	0-39	1.02 (0.94 - 1.10)	1.04 (0.95 - 1.14)	0.95 (0.85 - 1.06)	**0.88* (0.78 - 1.00)**
		40-49	0.97 (0.89 - 1.05)	0.98 (0.89 - 1.08)	0.99 (0.88 - 1.11)	0.94 (0.82 - 1.08)
		50-59	0.96 (0.92 - 1.00)	0.98 (0.93 - 1.02)	**0.93* (0.88 - 0.99)**	**0.90** (0.85 - 0.97)**
		60-69	**0.95** (0.92 - 0.98)**	0.98 (0.95 - 1.02)	**0.96* 0.92 - 1.00)**	**0.93** 0.89 - 0.97)**
		70-79	0.97 (0.92 - 1.02)	0.96 (0.91 - 1.02)	0.93 0.87 - 1.00)	0.93 (0.86 - 1.01)
		80+	**0.96* (0.92 - 0.99)**	**0.95* (0.92 - 1.00)**	**0.94* (0.90 - 0.99)**	1.00 (0.95 - 1.06)
		All	0.96 (0.91 - 1.02)	0.97 (0.91 - 1.05)	0.95 (0.87 - 1.03)	0.94 (0.85 - 1.03)
Colorectal	Male	0-69	0.95 (0.80 - 1.12)	0.96 (0.79 - 1.16)	0.94 (0.75 - 1.18)	0.96 (0.74 - 1.24)
		70+	0.92 (0.84 - 1.00)	**0.89* (0.80 - 0.99)**	**0.87* (0.77 - 0.98)**	0.89 (0.78 - 1.02)
		All	0.93 (0.80 - 1.08)	0.92 (0.77 - 1.10)	0.90 (0.74 - 1.10)	0.92 (0.73 - 1.16)
	Female	0-69	0.93 (0.78 - 1.11)	0.83 (0.68 - 1.02)	0.82 (0.65 - 1.04)	0.77 (0.59 - 1.00)
		70+	**0.83*** (0.75 - 0.92)**	**0.86* (0.76 - 0.98)**	**0.85* (0.74 - 0.98)**	**0.82* (0.70 - 0.97)**
		All	0.87 (0.73 - 1.04)	0.85 (0.69 - 1.05)	0.84 (0.66 - 1.06)	0.80 (0.61 - 1.05)
Lung	Male	0-69	1.00 (0.85 - 1.17)	0.97 (0.81 - 1.16)	0.94 (0.77 - 1.15)	0.96 (0.77 - 1.20)

		70+	0.96<br>(0.89 - 1.05)	0.95<br>(0.86 - 1.04)	**0.90***<br>**(0.81 - 1.00)**	0.92<br>(0.82 - 1.03)
		All	0.98<br>(0.85 - 1.13)	0.96<br>(0.82 - 1.12)	0.92<br>(0.77 - 1.10)	0.94<br>(0.77 - 1.14)
	Female	0-69	1.02<br>(0.87 - 1.20)	0.97<br>(0.82 - 1.16)	0.96<br>(0.80 - 1.16)	0.91<br>(0.74 - 1.13)
		70+	1.05<br>(0.94 - 1.17)	1.06<br>(0.94 - 1.20)	1.03<br>(0.90 - 1.19)	1.06<br>(0.90 - 1.25)
		All	1.03<br>(0.88 - 1.21)	1.01<br>(0.85 - 1.22)	1.00<br>(0.81 - 1.22)	0.98<br>(0.78 - 1.24)
Prostate	Male	0-69	**0.87***<br>**(0.75 - 1.00)**	0.88<br>(0.74 - 1.04)	0.87<br>(0.71 - 1.07)	0.86<br>(0.68 - 1.09)
		70+	**0.87*****<br>**(0.80 - 0.93)**	**0.89****<br>**(0.82 - 0.97)**	**0.87****<br>**(0.78 - 0.96)**	**0.88***<br>**(0.78 - 0.99)**
		All	**0.87***<br>**(0.76 - 0.99)**	0.89<br>(0.76 - 1.04)	0.87<br>(0.72 - 1.05)	0.87<br>(0.70 - 1.08)
Breast	Female	0-69	0.94<br>(0.86 - 1.02)	1.03<br>(0.94 - 1.14)	0.99<br>(0.89 - 1.11)	0.99<br>(0.87 - 1.12)
		70+	0.95<br>(0.87 - 1.04)	0.95<br>(0.86 - 1.05)	0.94<br>(0.83 - 1.06)	0.99<br>(0.86 - 1.14)
		All	0.94<br>(0.85 - 1.04)	1.00<br>(0.89 - 1.12)	0.97<br>(0.85 - 1.11)	0.99<br>(0.85 - 1.16)

Significant values ($p < 0.05$) are shown in bold. Levels of significance are additionally indicated by asterisks: *** $p < 0.001$, ** $p < 0.01$, * $p < 0.05$.

Supplementary Table 3 Observed cancer cases from 2020 to 2023 compared to expected cases under two assumptions: a) constant age-specific incidence rates based on the 2017-2019 reference period, and b) projected age-specific incidence trends extrapolated from 2015 to 2019

Site	Year	Observed cases	Difference to reference	Difference to projection
Total	2020-2023	258,157	-14,214	-19,525
	2020	63,948	-3,018	-3,773
	2021	64,885	-2,791	-3,914
	2022	64,208	-4,256	-5,766
	2023	65,116	-4,149	-6,073
Colorectal	2020-2023	26,701	-4,533	-3,496
	2020	6,785	-881	-732
	2021	6,720	-1,046	-824
	2022	6,630	-1,229	-933
	2023	6,566	-1,377	-1,008
Lung	2020-2023	24,294	-329	-709
	2020	6,107	+64	+11
	2021	6,060	-51	-127
	2022	5,972	-219	-325
	2023	6,155	-123	-267
Prostate	2020-2023	39,264	+647	-5,788
	2020	8,935	-520	-1,382
	2021	9,665	+84	-1,248
	2022	10,030	+314	-1,527
	2023	10,634	+769	-1,630
Breast	2020-2023	38,484	-1,934	-1,014
	2020	9,262	-715	-581
	2021	9,833	-215	-18
	2022	9,580	-568	-307
	2023	9,809	-435	-108

Supplementary Table 4 Data source information for federal state cancer incidence data and incidence estimates from other countries

Federal State / Country	Source
Bavaria (GER)	Krebsregister Bayern, Datenbankanfrage<br>Krebsregister: Datenbankabfrage<br>Data as of 2024/10/25
Bremen (GER)	Zeitverlauf Raten in Bremen, Bremer Krebsregister<br>https://www.krebsregister.bremen.de/<br>Data as of 2023/05/24
Hamburg (GER)	Datenbankexport Hamburg, Hamburgisches Krebsregister<br>https://www.hamburg.de/krebsregister/<br>Data as of 2024/07/01
Lower Saxony (GER)	Zeitverlauf Raten in Niedersachsen, Epidemiologisches Krebsregister Niedersachsen, http://www.krebsregister-niedersachsen.de/daten/interaktiver-bericht<br>Data as of 2024/12/17
North Rhine-Westphalia (GER)	Zeitverlauf Raten in Nordrhein-Westfalen, Landeskrebsregister Nordrhein-Westfalen, https://www.landeskrebsregister.nrw/krebs-in-nrw/online-jahresbericht/<br>Data as of 2024/07/19
Saarland (GER)	Krebsregister Saarland. Interaktive Datenbank zu Inzidenz und Mortalität von Krebserkrankungen im Saarland 1970-2022. Saarbrücken 2024.<br>https://krebsregister.saarland.de<br>Data as of 2024/07/01
Schleswig-Holstein (GER)	Zeitverlauf Raten in Schleswig-Holstein, Krebsregister Schleswig-Holstein<br>https://www.krebsregister-sh.de<br>Data as of 2025/01/06
Belgium	Belgian Cancer Registry<br>Drawing Module<br>Drawing module | Belgian Cancer Registry
Netherlands	Netherlands Cancer Registry (NCR), Netherlands Comprehensive Cancer Organisation (IKNL), derived via www.iknl.nl/en/ncr/ncr-data-figures [2025/02/27]
Denmark<br>Finland<br>Iceland<br>Norway<br>Sweden	Larønningen S, Arvidsson G, Bray F, Dahl-Olsen ED, Engholm G, Ervik M, Guðmundsdóttir EM, Gulbrandsen J, Hansen HL, Hansen HM, Johannesen TB, Kristensen S, Kristiansen MF, Kønig SM, Lam F, Laversanne M, Lydersen LN, Malila N, Mangrud OM, Miettinen J, Pejicic S, Petterson D, Skog A, Steig BÁ, Tian H, Aagnes B, Storm HH (2024). NORDCAN: Cancer Incidence, Mortality, Prevalence and Survival in the Nordic Countries, Version 9.4 (29.08.2024). Association of the Nordic Cancer Registries. Cancer Registry of Norway. Available from: https://nordcan.iarc.fr/<br><br>Engholm G, Ferlay J, Christensen N, Bray F, Gjerstorff ML, Klint A, Køtlum JE, Olafsdóttir E, Pukkala E, Storm HH (2010). NORDCAN – a Nordic tool for cancer information, planning, quality control and research. Acta Oncol. 49(5):725–36. https://doi.org/10.3109/02841861003782017 PMID:20491528

GER=Germany

Supplementary Table 5 Standardized incidence ratio between the mean age-standardized incidence for total cancer (ICD-10 C00-C97 without C44) in the years 2017-2019 and the age-standardized incidence in the respective year. Based on publicly available incidence data from cancer registries in German federal states and other countries.

Federal State / Country	Sex	Standardized incidence ratio (95% confidence interval) between the specific year and the mean incidence in 2017-2019		
		2020	2021	2022
Baden-Württemberg (GER)	Male	0.95	0.95	0.94
Bavaria (GER)	Male	0.97	0.97	0.99
Bremen (GER)	Male	0.97	0.94	x
Hamburg (GER)	Male	1.02	1.04	0.99
Lower Saxony (GER)	Male	0.97	0.95	0.95
North Rhine-Westphalia (GER)	Male	0.94	0.97	0.97
Saarland (GER)	Male	0.95	0.97	0.93
Schleswig-Holstein (GER)	Male	0.98	0.99	0.95
Belgium	Male	0.94	1.02	1.02
Denmark	Male	0.95	0.98	0.95
Finland	Male	0.97	0.98	0.98
Iceland	Male	0.99	1.04	1.07
Netherlands	Male	0.93	0.97	0.97
Norway	Male	0.97	0.98	0.99
Sweden	Male	0.91	0.96	0.99
Baden-Württemberg (GER)	Female	0.97	0.98	0.96
Bavaria (GER)	Female	0.95	0.97	0.96
Bremen (GER)	Female	0.99	0.96	x
Hamburg (GER)	Female	0.99	1.01	1.01
Lower Saxony (GER)	Female	0.98	0.99	0.96
North Rhine-Westphalia (GER)	Female	0.97	0.98	0.97
Saarland (GER)	Female	0.91	1.02	0.96
Schleswig-Holstein (GER)	Female	1.01	1.03	1.00
Belgium	Female	0.96	1.02	1.01
Denmark	Female	0.96	0.97	0.97
Finland	Female	0.96	1.00	0.99
Iceland	Female	0.99	1.03	1.01
Netherlands	Female	0.93	1.01	0.98
Norway	Female	0.97	1.02	1.03
Sweden	Female	0.95	1.02	0.98

GER=Germany, x = no estimates available

Supplementary Figure 1 Monthly age-standardized incidence (per 100.000 person-years) for colorectal (A), lung (B), prostate (C), and female breast cancer (D) by sex (men in grey, women in pink).

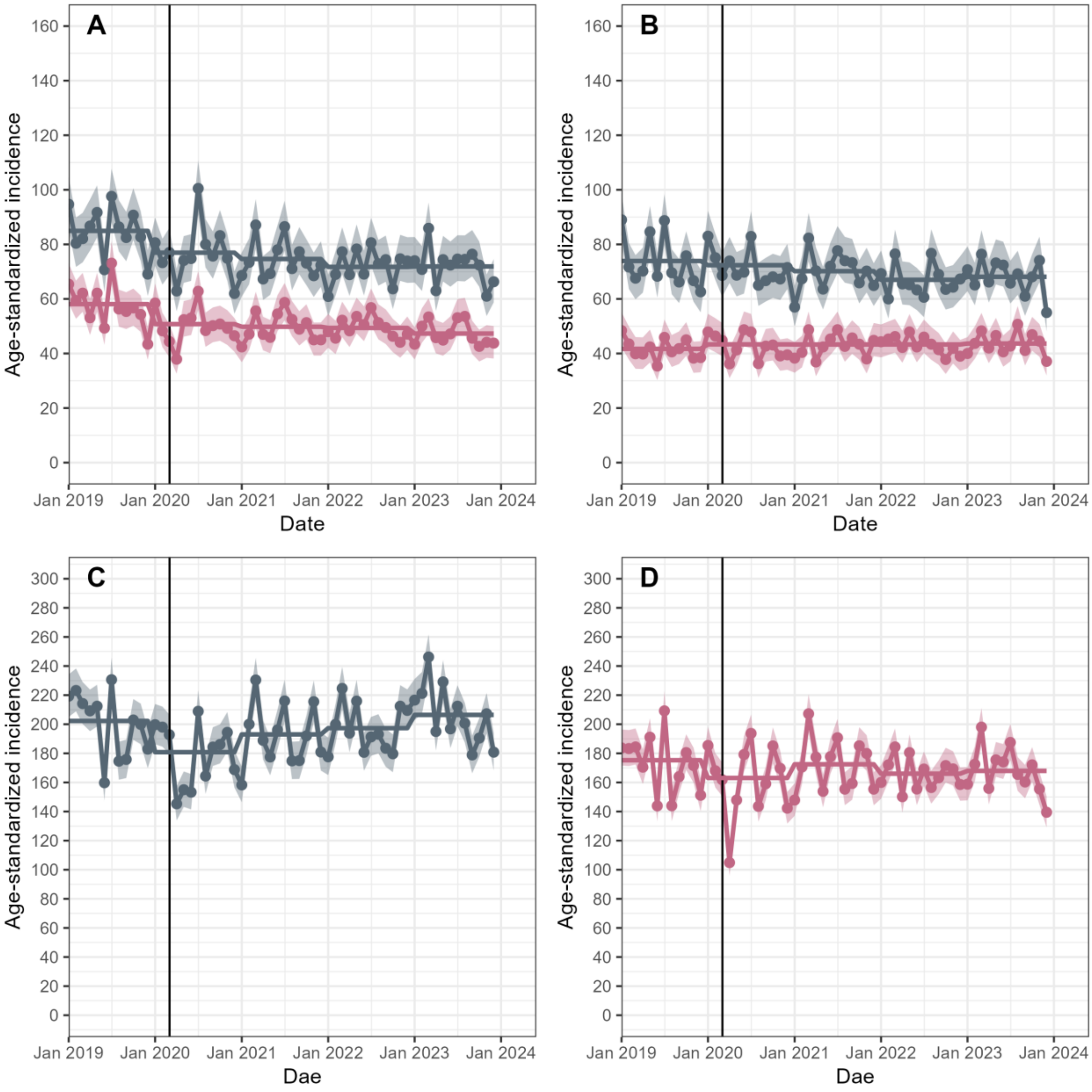

Supplementary Figure 2 Age-standardized (Europe 1976) total cancer incidence (ICD-10 C00-C97 without C44) in men (A) and women (B) by federal state in Germany starting in 2015 (restricted to registries with publicy available data, incidence per 100.000 person-years)

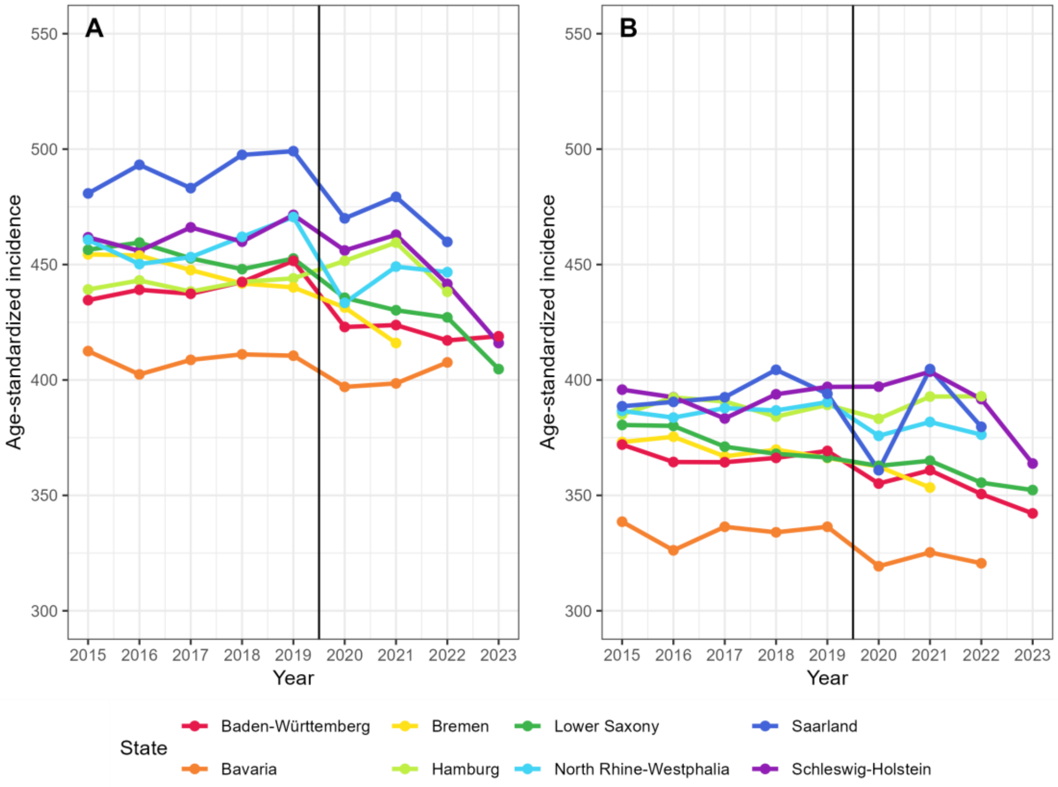

Supplementary Figure 3 Age-standardized (Europe 1976) total cancer incidence (ICD-10 C00-C97 without C44) in men (A) and women (B) by country starting in 2015 (incidence per 100,000 person-years)

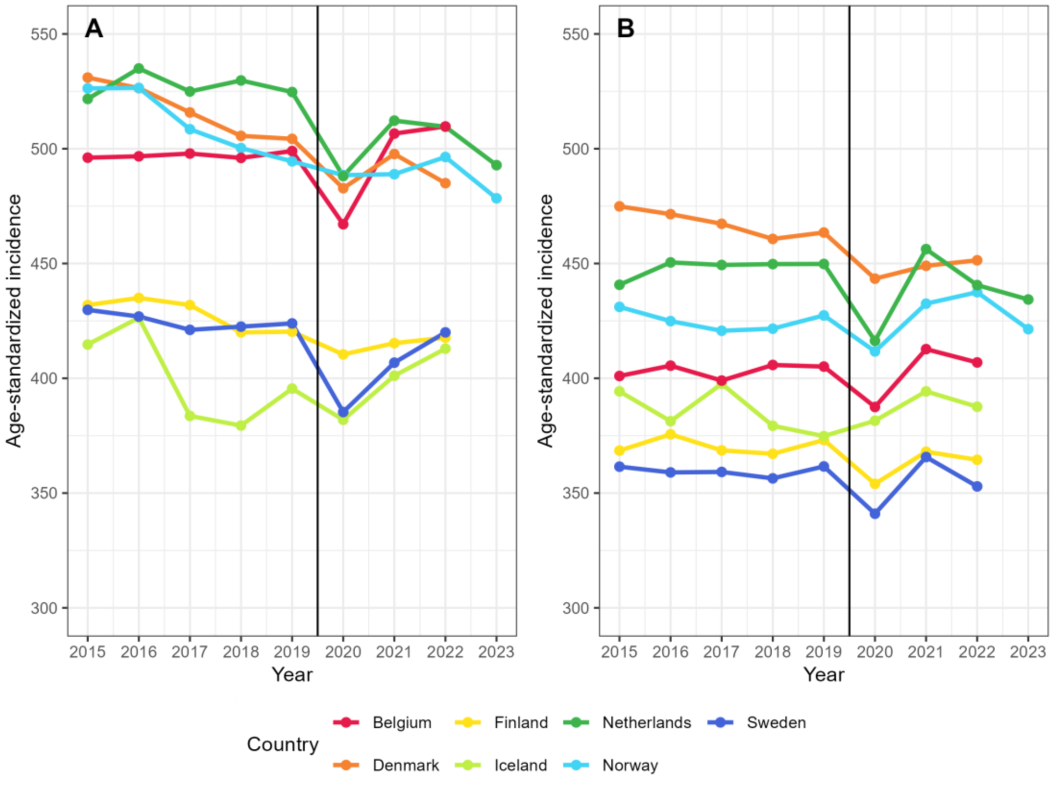

Supplementary Figure 4 Federal states in Germany. Baden-Württemberg is highlighted in dark blue.

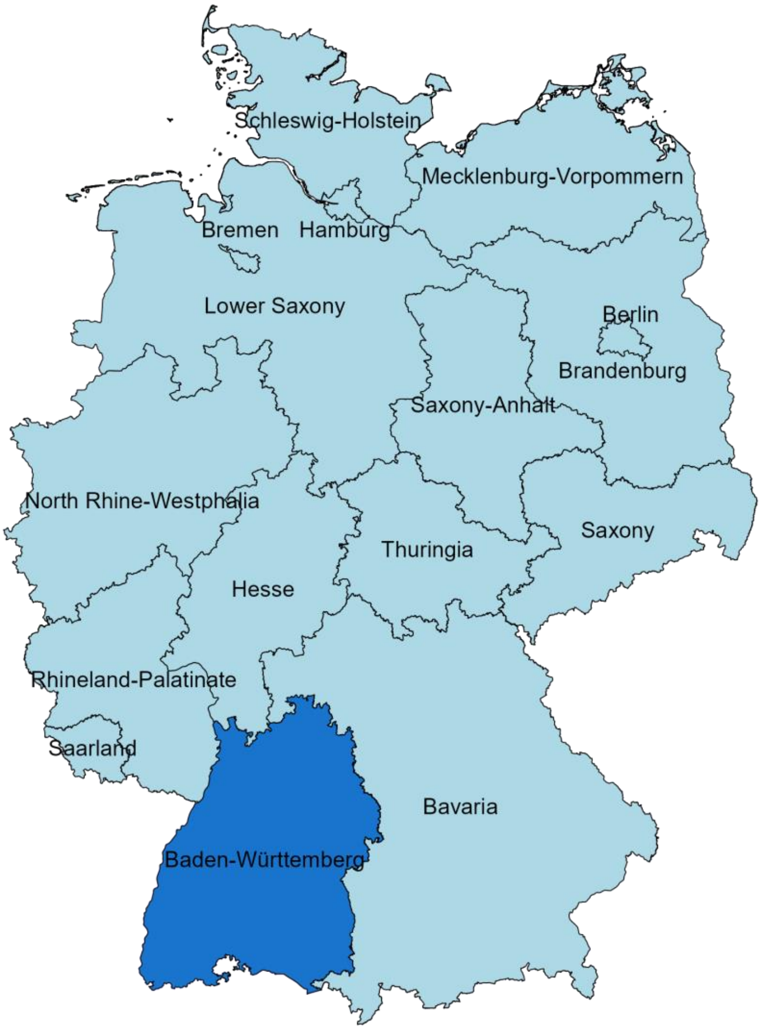

Supplement: Supplementary file 1 — Supplementary Material 1 [file 432_2025_6349_MOESM1_ESM.pdf]
